# Multi-omics analyses inform mechanisms of immunotherapy response in pancreatic cancer

**DOI:** 10.3389/fimmu.2025.1673098

**Published:** 2025-10-02

**Authors:** Mengyao Wu, Ge Li, Yecheng Li, Kai Chen, Mengdan Xu, Dapeng Li, Caihua Xu, Meng Shen, Wei Li, Jinming Cao

**Affiliations:** ^1^ Department of Oncology, the First Affiliated Hospital of Soochow University, Suzhou, China; ^2^ Department of Urology Surgery, Renji Hospital Affiliated to Shanghai Jiaotong University School of Medicine, Shanghai, China; ^3^ Department of General Surgery, the Second Affiliated Hospital of Soochow University, Suzhou, China; ^4^ Department of Nuclear Medicine, the First Affiliated Hospital of Soochow University, Suzhou, China; ^5^ State Key Laboratory of Radiation Medicine and Protection, Soochow University, Suzhou, China

**Keywords:** pancreatic cancer, immunotherapy, prognosis, multi-omics analysis, WTSS

## Abstract

**Introduction:**

Pancreatic ductal adenocarcinoma (PDAC) continues to exhibit resistance to immunotherapy. In this study, we evaluated the efficacy of combining immunotherapy with chemotherapy for the treatment of advanced pancreatic cancer. Additionally, we employed a multimodal analytical approach to elucidate the immune landscape and conduct transcriptomic profiling in PDAC.

**Methods:**

A retrospective analysis was conducted on the clinical data of 52 patients diagnosed with advanced PDAC who underwent a combined treatment regimen of immunotherapy and chemotherapy. The study evaluated the objective response rate (ORR), disease control rate (DCR), and progression-free survival (PFS). To characterize the immune landscape in treatment-naive pancreatic ductal adenocarcinoma (PDAC) tumors and in the systemic circulation, flow cytometry, multiplex immunohistochemistry (mIHC), and whole transcriptome sequencing were employed.

**Results:**

The study reported an ORR of 32.7%, a DCR of 67.3%, and a 6-month PFS rate of 38.5%, with a median PFS of 5.5 months. Patients treated with a combination of immunotherapy and gemcitabine achieved the longest PFS. The first-line treatment cohort exhibited a significantly higher DCR (79.3% vs. 52.2%, P = 0.038) and a longer median PFS (6.6 vs. 3.5 months, P = 0.032) compared to the second-line treatment cohort. The efficacy of treatment varied depending on the drug combinations used. Flow cytometry analysis revealed a greater frequency of CD45- CD64+ cells in the peripheral blood of patients with progressive disease (PD) compared to those with a partial response (PR). Multiplex immunofluorescence (MIF) analysis indicated an increased intratumoral infiltration of CD8+ T cells and CD137+ CD8+ T cells in patients with PR. Whole transcriptome sequencing (WTSS) identified key genes involved in immune regulation, signal transduction, and digestive function. Hemopexin (HPX) and regulatory factor X-associated protein (RFXAP) were upregulated in PR patients and showed a positive correlation with survival, whereas Interleukin-6 (IL-6) expression was linked to poor prognosis.

**Conclusions:**

These findings indicate that immunochemotherapy shows potential for the treatment of advanced PDAC. Our study elucidates the immune landscape associated with PDAC and provides critical insights for the identification of prospective therapeutic targets, which could guide the development of innovative combination immunotherapy strategies.

## Introduction

PDAC ranks as the third leading cause of cancer-related mortality among men and women combined in 2024, exhibiting lowest 5–year survival rate for merely 13% of all cancer patients (1). Metastatic PDAC presents an even bleaker prognosis, with a 5–year survival rate of only 3%, ranking among the poorest outcomes across common malignancies. Factors contributing to this dismal prognosis include late clinical presentation, a lack of effective screening methods, and the development of resistance to chemoradiotherapy in certain patient subgroups. Although combination chemotherapy has demonstrated efficacy in tumor control and clinical stabilization, standard regimens such as gemcitabine plus nab-paclitaxel (AG) and FOLFIRINOX (oxaliplatin, irinotecan, fluorouracil and leucovorin) are limited by transient response durability and considerable toxicity (2). Consequently, there is a critical need to investigate novel therapeutic strategies for this disease.

Immunotherapy has attracted considerable interest for its potential in treating pancreatic cancer, the therapeutic efficacy is influenced by various factors (3). However, immune checkpoint inhibitors (ICIs) have exhibited limited clinical activity in PDAC patients, likely due to the highly complex and immunosuppressive tumor microenvironment (4). Monotherapy targeting programmed cell death protein 1 (PD-1), programmed death-ligand 1 (PD-L1), or cytotoxic T-lymphocyte-associated protein 4 (CTLA-4) has shown minimal efficacy (ORR < 5%) in advanced PDAC, even among patients with positive PD-L1 expression, a biomarker associated with favorable responses in other cancers (5–7). Emerging evidence suggests that combining anti-PD-1/PD-L1 agents with chemotherapy, targeted therapy, or other immunotherapies may yield more favorable outcomes (8, 9). A major ongoing challenge is the identification of patients most likely to benefit from ICI-based treatment.

This study conducted an analysis of patients with advanced PDAC who received ICIs therapy at our institution, with a focus on investigating potential biomarkers that could be utilized for predicting the efficacy and prognosis of immunotherapy. Using advanced molecular profiling techniques including mIF and WTSS, we characterized immune landscapes to identify novel features associated with treatment response. A comprehensive understanding of the immune contexture of PDAC and its regulatory mechanisms is essential for the discovery of new predictive biomarkers and therapeutic targets, which may ultimately facilitate the development of personalized treatment strategies.

Our study highlights the potential benefits of combining immunotherapeutic agents with conventional treatments and provides a wealth of hypothesis-generating data intended to benefit the broader PDAC research community.

## Methods

### Patients and samples

This study conducted a single-center analysis of patients with advanced pancreatic cancer who received ICI-based combination therapies at the oncology department of the First Affiliated Hospital of Soochow University from January 2021 to December 2023. The inclusion criteria for study participants were as follows: 1. Pathological diagnosis of PDAC confirmed through surgical pathology slides or needle biopsies; 2. Stage IV disease according to the 8th edition of the TNM staging criteria for pancreatic cancer published by the American Joint Committee on Cancer; 3. Received anti-PD-1/PD-L1 therapy at least twice and had a post-baseline computed tomography scan; 4. Eastern cooperative oncology group (ECOG) score of ≤ 2 points. Exclusion criteria: 1. Patients with a history of prior immunotherapy with anti-PD-1/PD-L1; 2. Patients with previous or concurrent malignancies, except curatively treated skin basal cell carcinoma or cervical carcinoma *in situ*, with small gastric stromal tumors, as well as other early tumors after radical treatment; 3. Patients with a history of unstable angina pectoris, with arrhythmias (including QTcF ≥ 450 ms in men and ≥ 470 ms in women) requiring long-term antiarrhythmic drugs and New York Heart Association grade ≥ II cardiac insufficiency; 4. Patients with a history of immunodeficiency, or other acquired or congenital immunodeficiency diseases, or a history of organ transplantation; 5. Patients with infectious pneumonia, non-infectious pneumonia, interstitial pneumonia and others requiring corticosteroid use; 6. with history of chronic autoimmune diseases, such as systemic lupus erythematosus, with history of ulcerative enteritis, Crohn’s disease and other inflammatory bowel diseases, with history of irritable bowel syndrome and other chronic diarrheal diseases, with history of sarcoidosis or tuberculosis, with history of active hepatitis B, hepatitis C and HIV infection.

All patients were treated with anti-PD-1/PD-L1 therapy at standard doses (Pembrolizumab 200 mg, Camrelizumab 200 mg, Sintilimab 200 mg, Tislelizumab 200 mg, Penpulimab 200 mg, Serplulimab 300 mg, Adebrelimab 20 mg/kg every 3 weeks, or Envafolimab 150 mg every week), in combination with chemotherapy. Due to the limited sample size for any single agent, all PD-1/PD-L1 inhibitors were analyzed as a pooled class. All patients signed informed consent forms for immunochemotherapy before treatment, and evaluated every 6–8 weeks. Tumor responses were evaluated according to the response evaluation criteria in solid tumors (RECIST) version 1.1, which classified as complete response (CR), PR, stable disease (SD) and PD. The ORR was calculated as the sum of CR and PR, while the DCR was calculated as the sum of CR, PR, and SD. PFS was defined as the duration from the initiation of treatment to disease progression or death, with the last follow-up conducted in December 2023.

### Flow cytometry

2 mL of human peripheral whole blood was collected from EDTA vacuum anticoagulated blood collection tubes and allowed to rest horizontally at room temperature for up to 24 hours or disposal. 100 μL blood was added to a flow tube, followed by the appropriate volume of antibody, then incubated in darkness for 15 minutes at room temperature. Following this, 2 mL of 1× erythrocyte lysate was added to the whole blood and allowed to lyse in darkness for 10 minutes until the cell suspension became clear and transparent. The red blood cells were separated by centrifugation at 300 g for 5 minutes, with the supernatant being discarded. Subsequently, 2 mL of focusing fluid was added to each tube, mixed thoroughly, and centrifuged, abandoning the supernatant. 500 μL of focusing fluid was then added to each tube for flow cytometry detection. The primary antibodies utilized were HLA-DR FITC (BD, Cat. no. 665745), CD45 FITC (BD, Cat. no. 665752), CD45RA FITC (BD, Cat. no. 662840), CD4 FITC (BD, Cat. no. 340133), CD64 PE (BD, Cat. no. 652830), CD3 PE (BD, Cat. no. 663526), CD62L PE (BD, Cat. no. 304806), CD127 PE (BD, Cat. no. 664400), CD45 PerCP (BD, Cat. no. 664934), CD27 PerCP-CY55 (BD, Cat. no. 662839), CD3 PerCP (BD, Cat. no. 665748), CD8 PE-CY7 (BD, Cat. no. 664999), CD38 PE-CY7 (BD, Cat. no. 663494), PD-1 PE-CY7 (BD, Cat. no. 561272), CD4 APC (BD, Cat. no. 663498), CD19 APC (BD, Cat. no. 652804), CD25 APC (BD, Cat. no. 666484), CD3 APC H7 (BD, Cat. no. 663490), CD14 APC H7 (BD, Cat. no. 663492), CD28 APC CY7 (BD, Cat. no. 302966).

### Cytokine assay

The cytokine detection kit (Tianjin Kuangbo, Cat. no. 914002) was utilized for the assays. The serum was promptly separated within 4 hours after blood collection and allowed to clot at room temperature (20°C–25°C) for 30 minutes. Subsequently, the serum from the clear layer was centrifuged at 3000 RPM for 10–15 minutes. Microspheres cocktail and secondary antibody were added to a 1.5 ml EP tube and mixed using a vortex blender. 20 μL of standard or serum was then added and incubated for 2 hours at room temperature, avoiding light. Following this, 20 μL of SA-PE was added and shock incubated for 30 minutes at room temperature, away from light. Finally, 500 μL of 1× buffer was added and the mixture was centrifuged at 500 g for 5 min. The supernatant was discarded and 150 μL 1× buffer was added, vortexed and mixed before being tested on the machine with BeamDiag system.

### MIF assay

In order to investigate the expression and distribution of CD137, CD103, CD11c, and CD8 in PDAC, paraffin sections obtained from PDAC tissues were analyzed using mIF. The sections were deparaffinized by immersion in xylene for three times of 10 minutes each, followed by dehydration of pure ethanol for 5 minutes each. Antigen retrieval was conducted, followed by a natural cooling process. A Liquid Blocker PAP Pen was used to outline the tissue, and the sections were then incubated in 3% H_2_O_2_ for 25 minutes to block endogenous peroxidase activity. The blocking solution was removed, followed by the addition of the prepared primary antibody, and the slides were placed flat in a wet box for overnight incubation at 4°C. Subsequently, the slides were rinsed in PBS (pH 7.4) for 5 minutes and washed three times. The corresponding HRP labeled secondary antibody was then added and incubated at room temperature for 50 minutes. After another rinse in PBS (pH 7.4) and washing three times, the corresponding TSA was added and incubated at room temperature for 10 minutes in the dark. Following incubation, the slides were placed in TBST and washed on a decoloring shaker for 3 times. Tissue sections were subjected to antigen retrieval by being placed in a repair box filled with antigen repair buffer and heated in a microwave oven. The heating process involved initially using medium heat for 8 minutes, followed by a switch to medium-low heat for 7 minutes. Subsequently, a mixture of primary antibodies sourced from different origins was applied to the tissue sections, which were then incubated flat in a wet box at 4°C overnight. A combination of secondary antibodies was then applied and incubated at room temperature for 50 minutes in the absence of light. Finally, DAPI solution was added to the sections and incubated at room temperature for 10 minutes. The slides were incubated in PBS (pH 7.4) for 5 minutes and subsequently washed three times. Autofluorescence quencher B solution was then applied for 5 minutes, followed by a 10-minute wash with running water. Whole section imaging was performed using the 3DHISTECH Slide Converter. Images were analyzed using the ImageJ software. The primary antibodies utilized were CD137 (Abcam, Cat. no. ab252559, 1:1000), CD103 (Abcam, Cat. no. ab129202, 1:3000), CD11c (Servicebio, Cat. no. GB11059, 1:500), and CD8 (Servicebio, Cat. no. GB12068, 1:500).

### WTSS

WTSS was conducted on specimens obtained from three PDAC patients with significant tumor regression and three patients with significant tumor progression within our department. Prior to specimen collection, all patients had not undergone any treatments.

RNA Isolation and Library Preparation: Total RNA was extracted using the TRIzol reagent (Invitrogen, CA, USA) according to the manufacturer’s protocol. RNA purity and quantification were evaluated using the NanoDrop 2000 spectrophotometer (Thermo Scientific, USA). RNA integrity was assessed using the Agilent 2100 Bioanalyzer (Agilent Technologies, Santa Clara, CA, USA). The samples with qualified purity, quantity and integrity were used for subsequent library construction. Ribo-off rRNA Depletion Kit(vazyme, Nanjing, China)was used to remove ribosomal RNA, then the libraries were constructed using VAHTS Universal V6 RNA-seq Library Prep Kit according to the manufacturer’s instructions. The transcriptome sequencing and analysis were conducted by OE Biotech Co., Ltd. (Shanghai, China).

RNA sequencing analysis process: The libraries were sequenced on an llumina Novaseq 6000 platform and 150 bp paired-end reads were generated. The sequencing depth was 12 gigabases (Gb). Raw reads of fastq format were firstly processed using fastp and the low quality reads were removed to obtain the clean reads. The clean reads were mapped to the reference genome using HISAT2. The read counts of each gene were obtained by HTSeq-count and then FPKM of each gene was calculated. PCA analysis were performed using R (v 3.2.0) to evaluate the biological duplication of samples. Differential expression analysis was performed using the DESeq2. The resulting p-values were adjusted for multiple testing using the default Benjamini-Hochberg (FDR) method. Q value < 0.05 and foldchange > 2 or foldchange < 0.5 was set as the threshold for significantly differential expression gene (DEGs). Hierarchical cluster analysis of DEGs was performed using R (v 3.2.0) to demonstrate the expression pattern of genes in different groups and samples. Based on the hypergeometric distribution, GO, KEGG pathway, Reactome and WikiPathways enrichment analysis of DEGs were performed to screen the significant enriched term using R (v 3.2.0), respectively. Gene Set Enrichment Analysis (GSEA) was performed using GSEA software.

lncRNA analysis: Differential expression analysis was performed using the DESeq2. Q value < 0.05 and foldchange > 2 or foldchange < 0.5 was set as the threshold for significantly differential expression lncRNA (DELs). Pearson’s coefficient was used to calculate the expression correlation between DELs and DEGs, the significant related lncRNA-gene pairs were screened with the criteria of p < 0.05 and | cor | > 0.8. GO, KEGG Pathway, Reactome and WikiPathways enrichment analysis were performed for genes significantly co-expressed by lncRNAs to predict the function of lncRNAs. The binding of lncRNA and miRNA was predicted by miranda (v 3.3a) software.

circRNA analysis: Find_circ (v 1.2) and CIRI2 software were used to identify the circRNA. The circRNA’s parent gene was annotated according to its genomic position, and Circbase (http://www.circbase.org/) and CIRCpedia were used to identify known circRNAs. RPB (junction reads per billion mapped reads) was used to quantify circRNA, and DEGseq was used to calculate the differential expression of circRNA. The p value was calculated by NB (negative binomial distribution test). Q value < 0.05 and foldchange > 2 or foldchange < 0.5 was set as the threshold for significantly differential expression circRNAs (DECs). Based on the hypergeometric distribution, GO, KEGG pathway, Reactome and WikiPathways enrichment analysis of the differential expression circRNA’s parent gene were performed to evaluate the function of circRNA’s parent genes. Pearson’s coefficient was used to calculate the expression correlation between DECs and DEGs, the significant related circRNA-gene pairs were screened with the criteria of p < 0.05 and |cor| > 0.8. GO, KEGG Pathway, Reactome and WikiPathways enrichment analysis were performed for genes significantly co-expressed by circRNAs to predict the function of circRNAs. The binding of circRNA and miRNA was predicted by miranda (v 3.3a) software.

### Statistical analysis

Baseline patient and tumor features were summarized by descriptive statistics. Median progression-free survival was estimated by the Kaplan-Meier method along with 95% CIs constructed using the SPSS20.0 software. χ^2^ tests were used for rate comparison. For the comparison of immune phenotypes between PR and PD patients, unpaired t test or Mann-Whitney test was used to compare two independent groups after normality test. Graphing was performed using GraphPad Prism 8 software. All tests were two-sided.

## Results

### Patient characteristics

A total of 52 patients diagnosed with locally advanced or metastatic pancreatic cancer underwent at least two cycles of anti-PD-1/PD-L1 therapy between January 1, 2021, and December 22, 2023 were enrolled. Baseline characteristics of the patients were summarized in **Table 1**. The median age of the cohort was 66 years (range 50–79 years), with 33 patients (63.5%) being male. The majority of patients (n = 48, 92.3%) had an ECOG performance status of 0–1. Of the total, 29 patients (55.8%) received PD-1/PD-L1 inhibitors as first-line treatment, while 23 patients (44.2%) were treated in the second line or beyond. The selection of immunotherapy and chemotherapy was also shown in **Table 1**. Treatment regimens included immunotherapy combined with gemcitabine-based chemotherapy (n = 28), fluorouracil-based chemotherapy (n = 18), or other regimens (n = 6).

**Table 1:** Patient characteristics.

Characteristic	N = 52
Age	
Median (range)	66 (50–79)
≥65, n (%)	31 (59.6)
Sex, *n* (%)	
Male	33 (63.5)
Female	19 (36.5)
Previous treatment regimens, *n* (%)	
1	29 (55.8)
≥2	23 (44.2)
Immunotheray received, *n* (%)	
Pembrolizumab	4 (7.7)
Camrelizumab	3 (5.8)
Sintilimab	5 (9.6)
Tislelizumab	12 (23.1)
Penpulimab	12 (23.1)
Serplulimab	5 (9.6)
Adebrelimab	1 (1.9)
Envafolimab	10 (19.2)
Chemotherapy received, *n* (%)	
gemcitabine	28 (53.8)
fluorouracil	18 (34.6)
others	6 (11.5)

### Chemotherapy combined with ICIs confer a preferable tumor response

**Figure 1:**
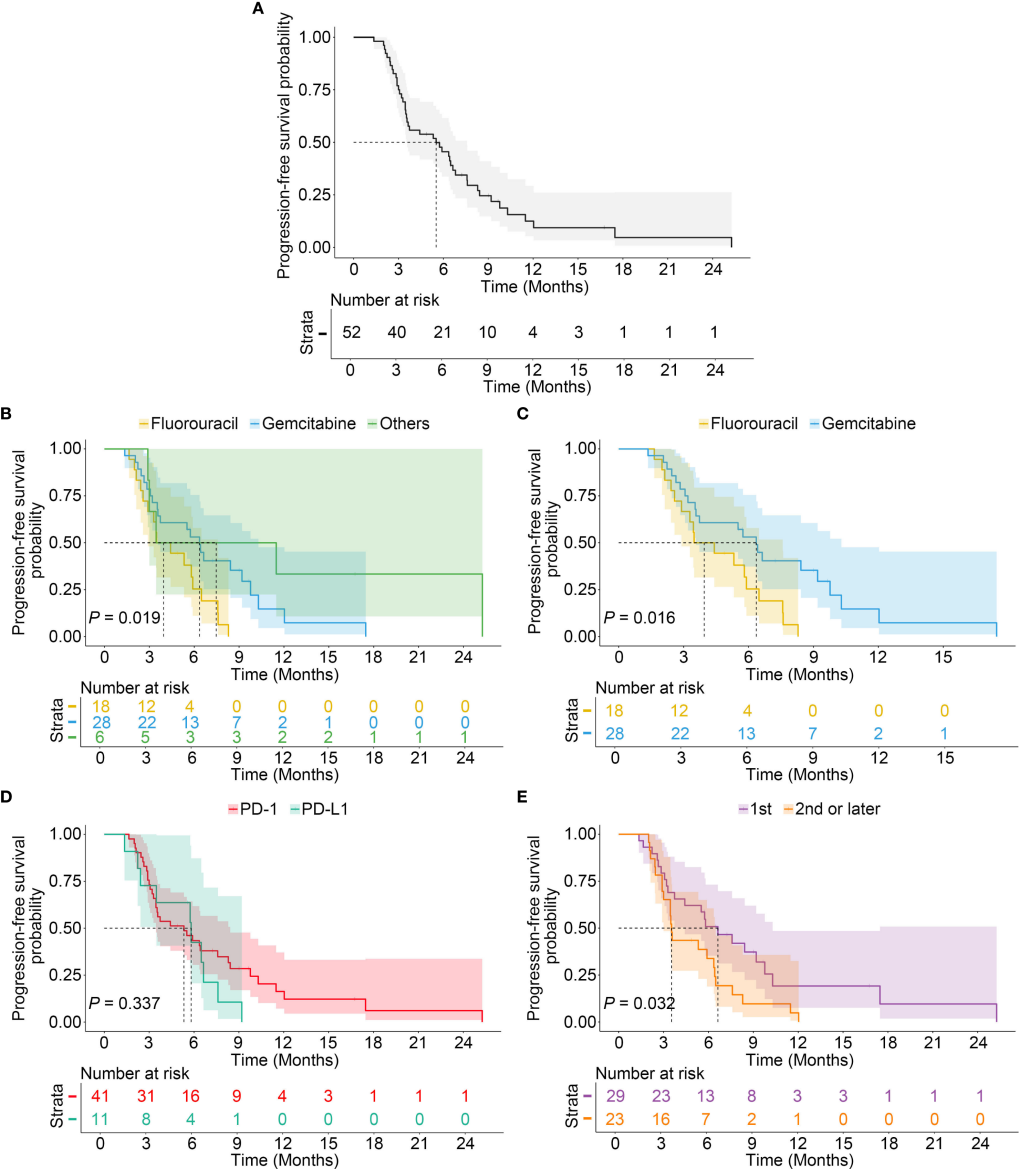
Kaplan–Meier estimates of progression-free survival. **(A)**. Kaplan–Meier curves depicting the PFS of all PDAC patients enrolled. **(B)**. Kaplan–Meier curves of subgroup analysis evaluated by the chemotherapy regimens. **(C)**. The median PFS of gemcitabine group was significantly longer than fluorouracil group. **(D)**. Kaplan–Meier curves of subgroup analysis evaluated by immunotherapy regimens. **(E)**. Kaplan–Meier curves of subgroup analysis evaluated by treatment lines.

In immuno-oncology clinical trials, conventional endpoints such as median PFS and median OS may initially provide misleading results due to the delayed therapeutic impact and modest cure rates. Based on insights from studies in melanoma, it is proposed that ORR may serve as a more suitable metric for observation. At the time of data cutoff for this analysis (Dec 22, 2023), 6 patients were still receiving immunotherapy, most patients had stable or responding disease at the onset of the study, although several patients were progressing to immunotherapy in combination with chemotherapy. Among the 52 patients, 17 (32.7%) achieved a PR, 18 (34.6%) were evaluated as SD, 17 (32.7%) experienced PD. The ORR was 32.7% (95% CI: 19.5–45.9%) and the DCR was 67.3% (95% CI: 54.1–80.5%) (**Table 2**). The 6-month PFS rate was 38.5% (95% CI: 24.8–52.1%), with a median PFS of 5.5 months (95% CI: 3.1–7.9 months) (**Figure 1A**).

**Table 2:** Clinical efficacy.

Variable	Total (*n* = 52)	Gemcitabine (*n* = 28)	Fluorouracil (*n* = 18)	Others (*n* = 6)	PD-1 (*n* = 41)	PD-L1 (*n* = 11)	1st-line (*n* = 29)	2nd or later line (*n* = 23)
Objective response								
No. of response	17	9	5	3	15	2	12	5
% of patients (95% CI)	32.7 (19.5-45.9)	32.1 (13.7-50.6)	27.8 (4.9-50.7)	50.0 (-7.5-107.5)	36.6 (21.2-52.0)	18.2 (-9.0-45.4)	41.4 (22.3-60.4)	21.7 (3.5-40.0)
Disease control								
No. of disease control	35	20	11	4	28	7	23	12
% of patients (95% CI)	67.3 (54.1-80.5)	71.4 (53.6-89.3)	61.1 (36.2-86.1)	66.7 (12.5-120.9)	68.3 (53.4-83.2)	63.6 (29.7-97.5)	79.3 (63.6-95.0)	52.2 (30.1-74.3)
Best overall response-no. (%)								
Partial response	17	9	5	3	15	2	12	5
Stable disease	18	11	6	1	13	5	11	7
Progression disease	17	8	7	2	13	4	6	11

Subgroup analysis based on chemotherapy regimens showed that in the gemcitabine group, the ORR was 32.1% (95% CI: 13.7–50.6%) and DCR was 71.4% (95% CI: 53.6–89.3%). In the fluorouracil group, ORR and DCR were 27.8% (95% CI: 4.9–50.7%) and 61.1% (95% CI: 36.2–86.1%) respectively. Patients receiving other regimens had an ORR of 50.0% (95% CI: -7.5–107.5%) and a DCR of 66.7% (95% CI: 12.5–120.9%). Immunotherapy combined with gemcitabine was associated with the longest PFS among all groups (*P* = 0.019) (**Figure 1B**). The median PFS was 6.4 months (95% CI: 5.0–7.8) in the gemcitabine group compared to 3.5 months (95% CI: 1.5–5.5) in the fluorouracil group (*P* = 0.016) (**Figure 1C**). After comparison of all relevant baseline clinical characteristics across the three treatment regimen groups, we found treatment lines correlated to chemotherapy regimen selection (**Supplementary Tables 1, 2**). Furthermore, there was no statistically significant difference in ORR and DCR between patients treated with anti-PD-1 versus anti-PD-L1 agents (36.6% vs. 18.2%, *P* = 0.428; 68.3% vs. 63.6%, *P* = 0.945). No significant difference was observed in PFS between PD1 and PD-L1 groups (5.3 vs. 5.8 months, *P* = 0.337, **Figure 1D**). First-line treatment was associated with a significantly higher DCR (79.3% vs. 52.2%, *P* = 0.038) and longer median PFS (6.6 vs. 3.5 months, *P* = 0.032) compared to second-line or later treatment (**Figure 1E**).

### Peripheral immune phenotype differs between response groups

**Figure 2:**
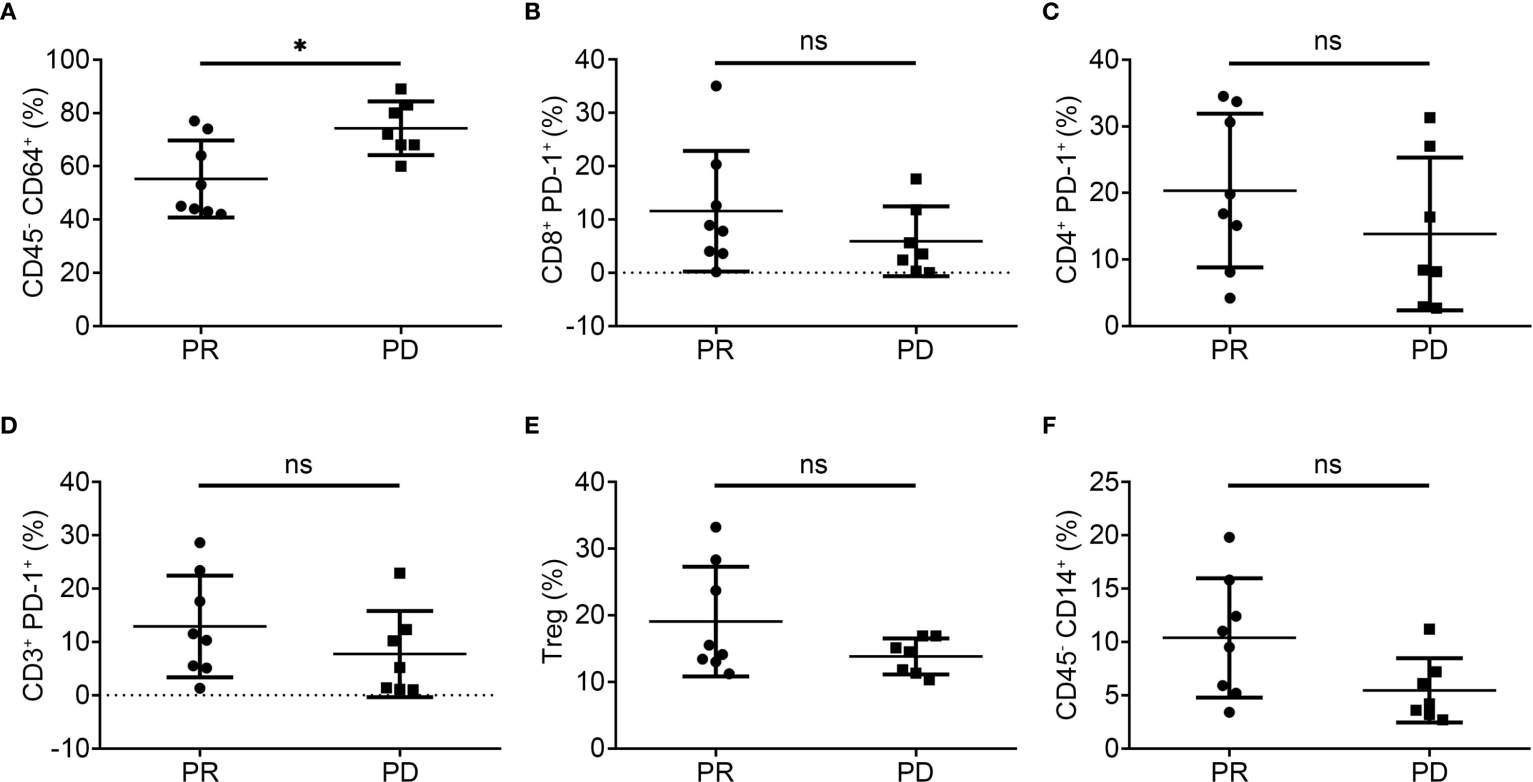
The immune phenotype of peripheral blood in PR and PD patients at baseline. **(A)**. Percentage of CD45^-^ CD64^+^ leukocytes in PR and PD patients. **(B)**. Percentage of CD8^+^ PD-1^+^ leukocytes in PR and PD patients. **(C)**. Percentage of CD4^+^ PD-1^+^ leukocytes in PR and PD patients. **(D)**. Percentage of CD3^+^ PD-1^+^ leukocytes in PR and PD patients. **(E)**. Percentage of Treg in PR and PD patients. **(F)**. Percentage of CD45^-^ CD14^+^ leukocytes in PR and PD patients. All data shown as the mean ± SD; *P < 0.05; ns, not significant.

Flow cytometry was performed on pre-treatment peripheral blood samples from 15 patients (8 PR, 7 PD) to investigate the correlation between immune phenotypes and patient prognosis. PD patients showed a significantly higher abundance of CD45^-^ CD64^+^ cells compared to PR patients (**Figure 2A**). No significant differences were observed in frequencies of CD8^+^ PD-1^+^, CD4^+^ PD-1^+^, CD3^+^ PD-1^+^ T cells, Tregs, or CD45^-^ CD14^+^ cells (**Figures 2B–F**). The same results went for CD8^+^ CD45RA^-^/CD8^+^, CD8^+^ CD28^+^/CD8^+^, CD8^+^ CD38^+^/CD8^+^, CD4^+^ CD45RA^-/^CD4^+^, CD4^+^ CD28^+^/CD4^+^, CD4^+^ CD38^+^/CD4^+^, CD45^+^ HLA-DR^+^, CD45RA^+^ CD62L^+^/CD4^+^, CD45dim CD3^-^ CD14^-^ CD27^-^ CD19^+^, CD45dim CD3^-^ CD14^-^ CD27^+^ CD19^+^, CD45dim CD3^-^ CD14^-^ CD27^+^ CD38^+^ CD19dim and CD8^+^ CD45RA^+^ CD62L^+^/CD8^+^ (**Supplementary Figures 1A–L**).

Simultaneously, plasma samples from a total of 26 patients were collected to assess cytokine levels at baseline. With 13 PR patients and 13 PD patients included in the study, we detected no differences of IL-1β, IL-2, IL-4, IL-5, IL-6, IL-8, IL-10, IL-12p70, IL-17A, IL-17F, IL-22, TNF-α, TNF-β or IFN-γ (**Supplementary Figures 2A–N**).

### Spatial immune infiltration analyzed by mIF

**Figure 3:**
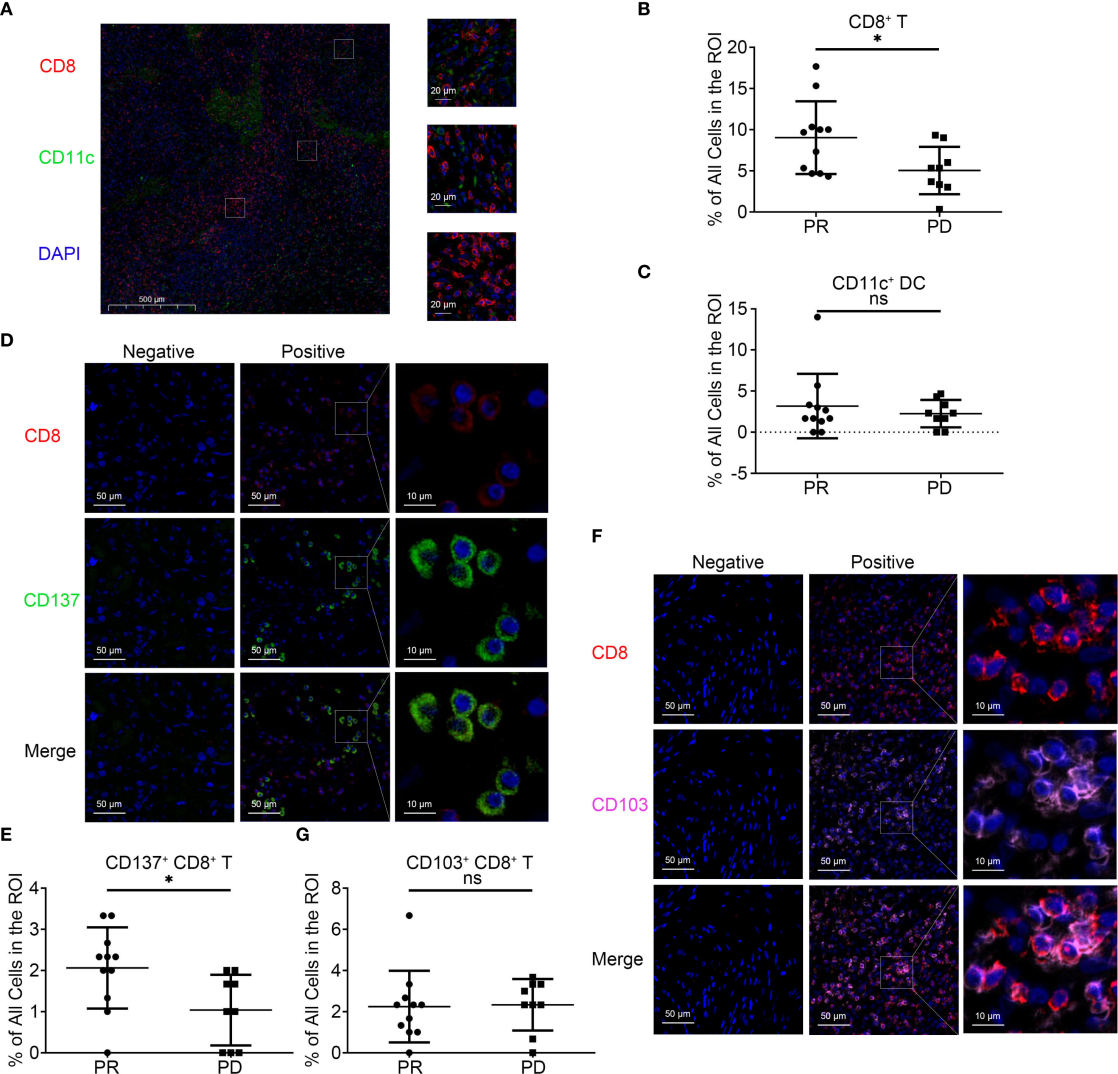
Multiplex immunohistochemistry of PDACs before immunotherapy. **(A)**. Representative images of mIF in PDAC. Cell markers, including CD8, CD11c and DAPI were labeled with different colors. **(B)**. Quantitation of CD8^+^ cells in PDAC tissue pretreatment. **(C)**. Quantitation of CD11c^+^ cells in PDAC tissue pretreatment. **(D)**. Representative images of mIF and quantitation of CD137^+^ CD8^+^ cells in PDAC tissue pretreatment. **(E)**. Quantitation of CD137^+^ CD8^+^ cells in PDAC tissue pretreatment. **(F)**. Representative images of mIF and quantitation of CD103^+^ CD8^+^ cells in PDAC tissue pretreatment. **(G)**. Quantitation of CD103^+^ CD8^+^ cells in PDAC tissue pretreatment. All data shown as the mean ± SD; *P < 0.05; ns, not significant.

MIF was applied to pre-treatment tumor tissues from 19 patients (10 PR, 9 PD). Regions of interest (ROIs) were specifically chosen within the pre-treatment tumor regions, with a minimum of three ROIs selected from each tumor specimen. CD8^+^ T cells and dendritic cells (DCs) were enriched in the tumor periphery (**Figure 3A**). PR patients exhibited significantly higher densities of intratumoral CD8^+^ T cells (**Figure 3B**) and CD137^+^ CD8^+^ T cells (**Figures 3D, E**). No significant differences were observed in CD11c^+^ DC infiltration (**Figure 3C**) or CD103^+^CD8^+^ T cell subsets (**Figures 3F, G**). Cross-presenting CD103^+^CD11c^+^ DCs were rarely detected.

### Transcriptomic signatures associated with treatment response

**Figure 4:**
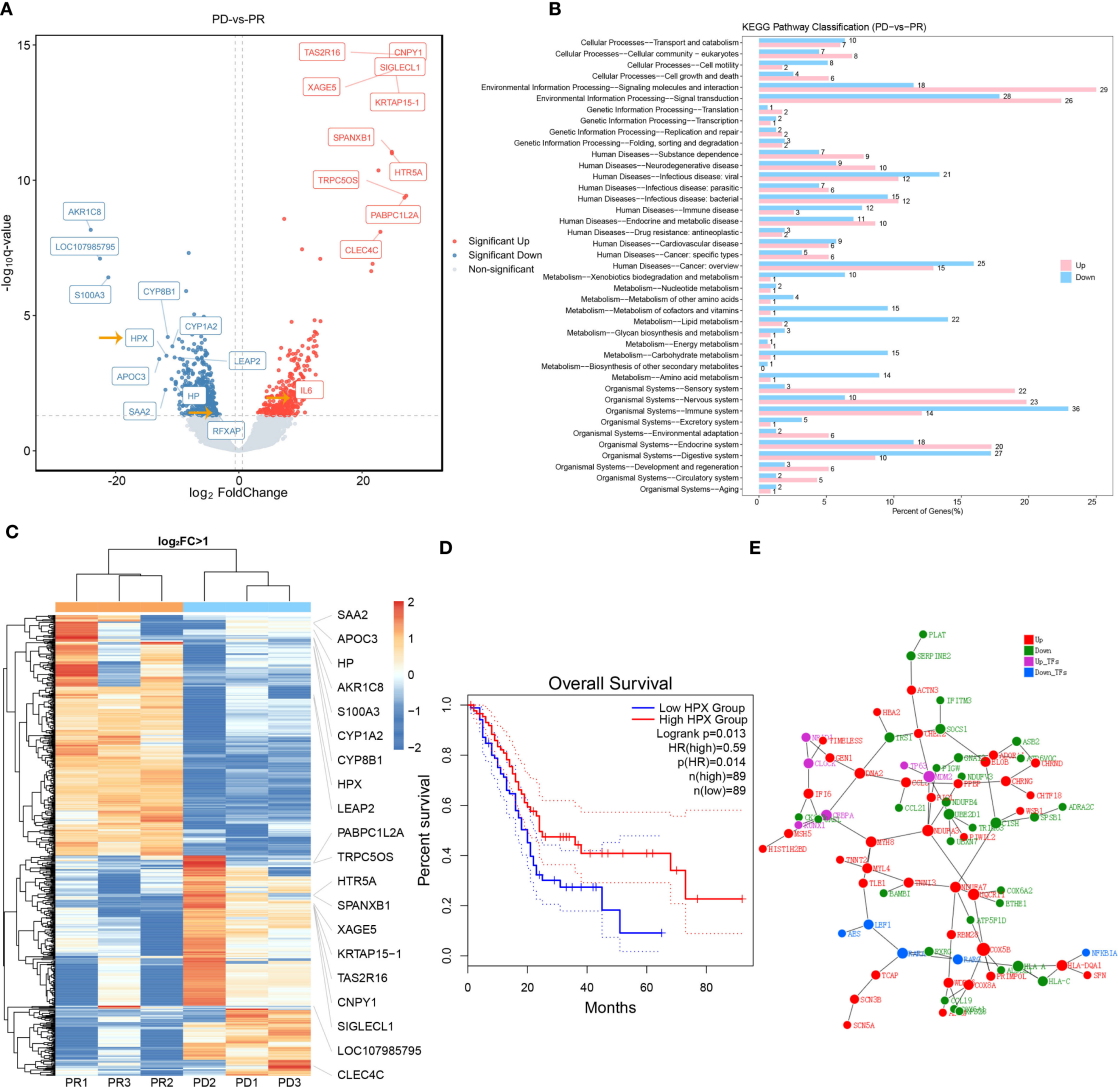
WTSS of DEGs between treatment responders and nonresponders. **(A)**. Volcano plot of DEGs between PR and PD patients. Cutoffs of absolute log2(fold change) >1 and q < 0.05 were used to identify significantly differentially expressed genes. Genes with non-significant differences are shown in gray, while significant differences are shown in red and green. **(B)**. KEGG pathway classification of DEGs. **(C)**. Cluster analysis of the DEGs between groups, red indicates relatively high expression genes and blue indicates relatively low expression genes. **(D)**. Kaplan–Meier curves depicting the relationship between HPX and survival in the TCGA PAAD database. **(E)**. Protein-protein interaction network analysis of the top 300 DEGs. Red represents up-regulated gene expression and green represents down-regulated gene expression. Purple represents that the differential gene is a transcription factor and its expression is up-regulated, and blue represents that the differential gene is a transcription factor and its expression is down-regulated.

Our study focused on identifying transcriptional predictors of response by utilizing WTSS to analyze differentially expressed genes between responders (PR) and non-responders (PD) (**Figure 4A**). A total of 746 up-regulated genes and 733 down-regulated genes were identified, with 9 genes found to be significantly overexpressed in the PR group: S100A3, APOC3, SAA2, HPX, CYP8B1, HP, CYP1A2, LEAP2, AKR1C8 (**Figure 4C**). The Cancer Genome Atlas (TCGA) PAAD database showed a positive correlation between HPX expression and survival (**Figure 4D**). In order to systematically identify differentially expressed pathways, KEGG pathway analysis was conducted (**Figure 4B**). The top enriched KEGG pathways for response-associated differentially expressed genes encompassed immune system, signal transduction, and digestive system. Subsequently, protein-protein interaction network analysis was conducted, ranking the interaction results of the top 300 differential genes based on their combined interaction score. A 3D interaction network was constructed by incorporating the annotation of the differential genes and transcription factors. The most closely associated down-regulated hub genes in the PD group included HLA-A, HLA-C and CCL19 (**Figure 4E**).

**Figure 5:**
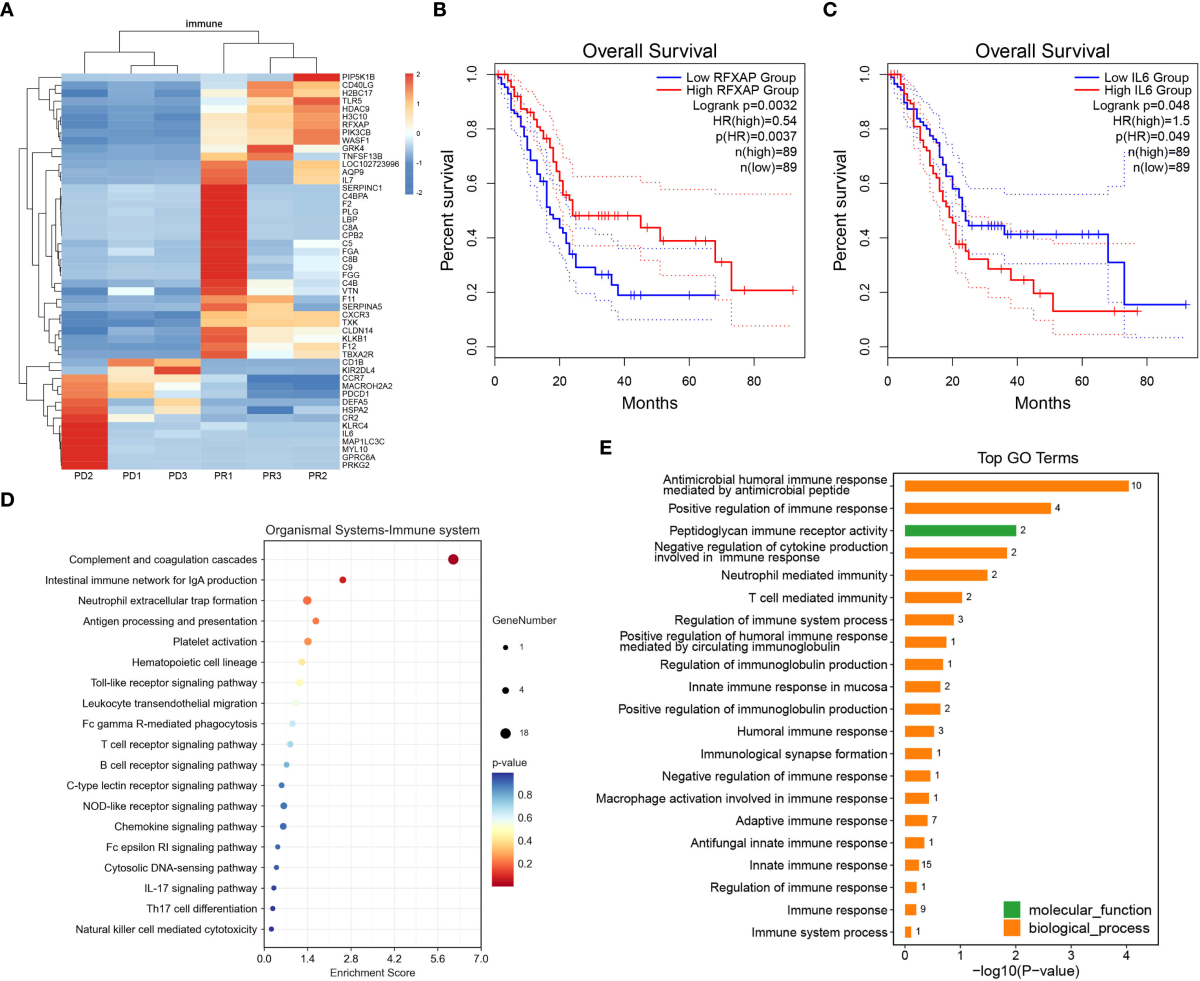
Transcriptomic features of representative immune-related genes. **(A)**. Cluster analysis of the representative immune-related DEGs between groups. Red indicates relatively high expression genes and blue indicates relatively low expression genes. Cutoffs of absolute log2(fold change) >1 and q < 0.05 were used to identify DEGs. **(B)**. Kaplan–Meier curves depicting the relationship between RXFAP and survival in the TCGA PAAD database. **(C)**. Kaplan–Meier curves depicting the relationship between IL6 and survival in the TCGA PAAD database. **(D)**. WikiPathways enrichment bubble plots of immune-related DEGs. **(E)**. GO enrichment analysis of immune-related DEGs.

Representative immune-related genes exhibiting differential expression in the PR and PD group were further explored (**Figure 5A**). Up-regulated immune-related genes in response positively group included PIP5K1B, CD40LG, H2BC17, TLR5, HDAC9, H3C10, RFXAP, PIK3CB, WASF1, GRK4, TNFSF13B, LOC102723996, AQP9, IL-7. Notably, high expression of RFXAP was correlated with longer OS (**Figure 5B**). Immune-related genes associated with resistance were identified as CD1B, KIR2DL4, CCR7, MACROH2A2, PDCD1, DEFA5, HSPA2, CR2, KLRC4, IL-6, MAP1LC3C, MYL10, GPRC6A, and PRKG2. Analysis using GEPIA2 revealed a negative correlation between IL-6 expression and prognosis in pancreatic cancer patients (**Figure 5C**). Additionally, WikiPathways enrichment bubble plots and GO enrichment analysis were utilized to investigate differentially expressed immune-related genes (**Figures 5D, E**), with the complement and coagulation cascades being identified as the most enriched pathway.

**Figure 6:**
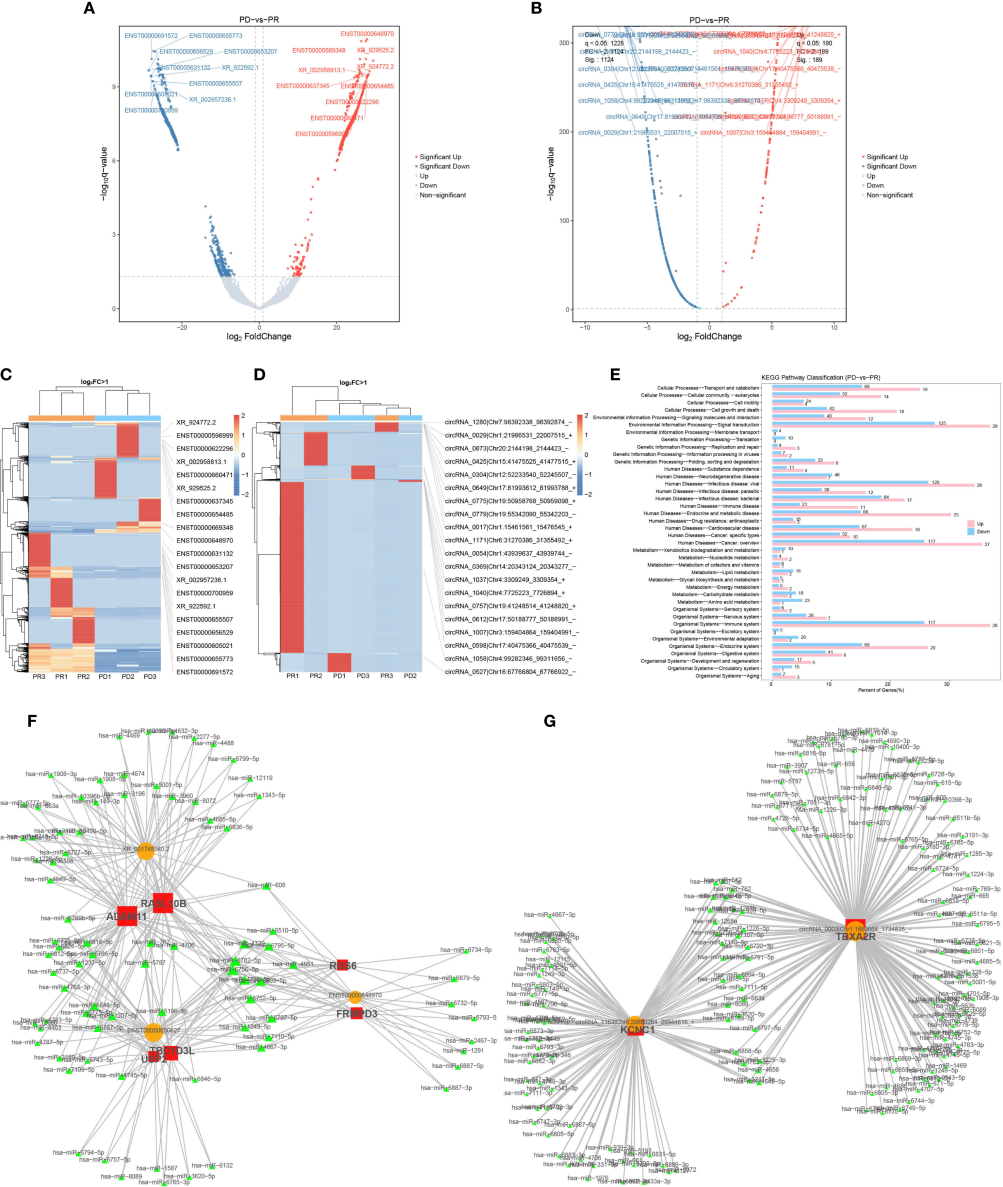
Differentially expressed lncRNA and circRNA between treatment responders and nonresponders. **(A)**. Volcano plot of differentially expressed lncRNA between PR and PD patients. Cutoffs of absolute log2(fold change) >1 and q < 0.05 were used to identify significantly differentially expressed lncRNA. Genes with non-significant differences are shown in gray, while significant differences are shown in red and green. **(B)**. Volcano plot of differentially expressed circRNA between PR and PD patients. **(C)**. Cluster analysis of differentially expressed lncRNA, red indicates relatively high expression lncRNA and blue indicates relatively low expression lncRNA. **(D)**. Cluster analysis of differentially expressed circRNA. **(E)**. KEGG pathway classification of top differentially expressed circRNA host genes. **(F)**. 200 mRNA-miRNA-lncRNA of the top 100 mRNA-lncRNA relationship pairs in the ceRNA analysis results were plotted. **(G)**. Network of 200 mRNA-miRNA-circRNA of the top 100 mRNA-lncRNA relationship pairs in the ceRNA analysis results.

Analysis of non-coding RNAs identified 870 up-regulated and 1104 down-regulated lncRNAs in PD patients (**Figures 6A, C**), as well as 189 up-regulated and 1124 down-regulated circRNAs (**Figures 6B, D**). Host genes of differentially expressed circRNAs were primarily involved in signal transduction, infectious disease, and immune system processes (**Figure 6E**). Due to limited expression data availability, miranda program was utilized to predict binding interactions between miRNA-lncRNA sequences, resulting in the identification of 27,203 miRNA-lncRNA pairs. Pearson test was used to calculate the correlation between different expressed mRNA and different expressed lncRNA. According to the role of mRNA-lncRNA in the competing endogenous RNA (ceRNA) relationship, a total of 169,087 mRNA-lncRNA with positive correlation were screened. Among these pairs, XR_001745340.2, ENST00000650627 and ENST00000648970 were found to interact with the highest number of miRNAs, corresponding to 6 mRNAs such as RASL10B, ADAM11, TBC1D3L, USP2, RGS6 and FRMPD3 (**Figure 6F**). The identical approach was employed in establishing the top mRNA-miRNA-circRNA network in the ceRNA analysis, a total of 126566 mRNA-circRNA pairs exhibiting positive correlation. Among these, only two mRNA-circRNA pairs, KCNC1-circRNA_1163 and TBXA2R-circRNA_0003, were found to interact with the highest number of miRNAs within the network (**Figure 6G**).

Taken together, the prominent mRNA, lncRNA, and circRNA identified through WTSS indicated the transcriptional correlates of response to ICIs in PDAC patients.

## Discussion

Despite recent advances in the understanding and treatment of pancreatic cancer, the prognosis for PDAC remains poor. Immunotherapy has shown limited efficacy in PDAC patients, underscoring the need for further research to better comprehend this complex disease and develop more effective treatment strategies.

The KEYNOTE-158 trial reported that patients with mismatch repair-deficient (dMMR) advanced cancers responded favorably to Pembrolizumab, with an ORR of 18.2% in the pancreatic cancer cohort. However, median PFS and overall survival (OS) were only 2.1 and 4.0 months, respectively (10). It should be noted that only approximately 2% of PDAC patients exhibit this molecular subtype. Similarly, a phase II trial evaluating Durvalumab (an anti-PD-L1 antibody) with or without Tremelimumab (an anti-CTLA-4 antibody) demonstrated limited activity, with an ORR of only 3.1% for combination therapy and 0% for monotherapy (11).

Chemotherapy remains the cornerstone treatment for most PDAC patients. Regimens such as AG, mFOLFIRINOX, and NALIRIFOX (liposomal irinotecan, oxaliplatin, fluorouracil, and leucovorin) are current options for metastatic PDAC patients with good performance status. The PRODIGE trial showed that FOLFIRINOX improved median OS to 11.1 months, median PFS to 6.4 months, and achieved an ORR of 31.6% (12). The phase III MPACT study demonstrated that AG treatment resulted in a DCR of 48% (with 23% ORR), median PFS of 5.5 months, and median OS of 8.5 months in treatment-naïve patients (13). More recently, the NAPOLI-3 trial showed that the NALIRIFOX regimen achieved a median PFS of 7.4 months and ORR of 41.8% (14). These data highlight the challenge of achieving sustained responses beyond 6 months with conventional chemotherapy, particularly in the Chinese population where access to newer agents like liposomal irinotecan remains limited.

Immunotherapy, particularly when combined with other treatment modalities, has demonstrated potential to induce tumor regression and improve OS in preclinical models of PDAC (15). Clinically, the combination of PD-1 inhibitors with chemotherapy has shown promising results. For example, a 2017 phase Ib/II trial evaluating Pembrolizumab in combination with AG in patients with metastatic pancreatic cancer reported a DCR of 100% (16). Further supporting this approach, studies of AG combined with either Pembrolizumab or Nivolumab in patients with locally advanced or metastatic pancreatic cancer showed ORR of 20% and 18%, median PFS of 9.1 and 5.5 months, and median OS of 15.0 and 9.9 months, respectively (17). In contrast, the combination of Ipilimumab and gemcitabine did not improve efficacy in previously treated PDAC patients, with an ORR of 14% and disease stabilization in 33% of patients (18).

Our study demonstrates that combining chemotherapy with immunotherapy resulted in an ORR of 32.7%, DCR of 67.3%, and median PFS of 5.5 months in 52 PDAC patients. Notably, first-line treatment achieved particularly encouraging outcomes with an ORR of 41.4%, DCR of 79.3%, and median PFS of 6.6 months. These outcomes surpassed results from most chemotherapy-only trials. Notably, patients receiving gemcitabine-based combinations exhibited the longest PFS, possibly due to gemcitabine’s ability to activate the host immune system, induce tumor cell apoptosis and enhance antigen presentation, thereby potentially overcoming immunosuppression and potentiating immunotherapy efficacy.

The success of immunotherapy in PDAC may hinge on appropriate patient selection and the identification of targets to transform the PDAC tumor microenvironment (TME) from an immunologically “cold” to an immunologically “hot” phenotype (19). While current biomarkers such as PD-L1 expression, tumor mutational burden, and microsatellite status have limited utility in PDAC (20), our comprehensive multi-omics analysis utilizing flow cytometry, mIF, and WTSS has identified several promising immune features associated with treatment response. We observed enhanced CD8^+^ T cell infiltration in responding patients, particularly a subset of CD137^+^ CD8^+^ T cells within the tumor microenvironment. These cells represent tumor antigen-specific T cells, with CD137 serving as a co-stimulatory receptor that promotes T cell survival and proliferation (21). CD137 expression is associated with cytotoxic effector T cell characteristics and improved survival. Research has shown that anti-CD137 agonist antibodies can augment the proliferation, memory, and activation status of effector T cells (22), suggesting that CD137 agonist therapy might enhance antitumor responses.

Moreover, efforts to improve the prognosis of pancreatic cancer patients should not only focus on enhancing T cell function but also on optimizing the intracellular environment of tumor cells. Recent studies have investigated gene expression in pancreatic tumors using single-cell RNA sequencing or high-content *in situ* hybridization (23–25). But these studies did not compare the tissue characteristics between patients responsive and non-responsive to immunotherapy. Only one study has reported the utilization of multi-omics approaches to characterize cellular states within and adjacent to tertiary lymphoid structures (TLS), spanning diverse spatial niches and pathological responses of combination neoadjuvant immunotherapies (26). Despite these efforts, the heterogeneity of PDAC at the mRNA level remains poorly understood. In this study, we utilized WTSS of tumor tissue to uncover a diverse genetic network among patients exhibiting different responses to ICIs based therapy. Pathway analysis highlighted involvement in immune regulation, signal transduction, and digestive function. Notably, HPX was overexpressed in responders and correlated with improved survival. The immune-related gene RFXAP exhibited up-regulation in the response group and was associated with prolonged OS, whereas the immunotherapy resistance gene IL-6 showed a negative association with the prognosis of pancreatic cancer patients.

HPX is a 57-kDa circulating glycoprotein synthesized in the liver with a high binding affinity for the iron-containing molecule heme, known for its pro-oxidant and inflammatory properties (27). Studies using HPX^^-^/^-^^ mouse models of prostate cancer have revealed more aggressive tumor phenotypes, suggesting a potential tumor-suppressive role for HPX (28). In liver hepatocellular carcinoma, high HPX expression was identified as a hub gene among cytotoxic CD8^+^ T cell-related markers and correlated significantly with improved patient survival (29). In contrast, elevated HPX levels have been linked to disease progression in colorectal cancer (30). The role of HPX in pancreatic cancer has not been thoroughly investigated, warranting further research.

The transcription factor RFXAP plays a critical role in regulating MHC class II molecules (31). Its deficiency can lead to impaired activation of CD4^+^ T lymphocytes, resulting in bare lymphocyte syndrome (32). In PDAC, RFXAP has been identified as a pivotal hub gene in cancer immunity, showing an inverse correlation with prognostic risk (33), reduced RFXAP expression is associated with advanced TNM stage and poorer patient prognosis. Furthermore, elevated RFXAP levels have been shown to interfere with DNA repair mechanisms and exacerbate DNA damage. Additionally, exosomes derived from pancreatic cancer cells have been found to suppress RFXAP expression, thereby reducing MHC II expression and promoting immune tolerance (34).

IL-6 is implicated in multiple facets of tumor progression, including tumor growth, angiogenesis, metastasis, metabolic reprogramming, therapy resistance, cachexia, and immune tolerance (35, 36). Within adaptive immunity, IL-6 inhibits antigen presentation by downregulating human leukocyte antigen (HLA)-DR and CD86 expression, enhancing arginase activity, and inducing immunosuppressive genes. These mechanisms collectively impair the activation of antigen-specific CD4^+^ T cells while promoting pro-tumorigenic regulatory T cell (Treg) and Th17 responses, ultimately fostering an immune-tolerant microenvironment (37). IL-6 has been established as a predictor of poor response to ICIs in various advanced cancers (38). This pleiotropic cytokine signals through membrane receptor complexes containing glycoprotein 130 (GP130), activating multiple downstream pathways (39). In PDAC, IL-6 accelerates cancer proliferation and metastasis via the miR-455-5p/IGF-1R axis (40), mediates gut microbiota-induced oncogenic effects through immune and inflammatory pathways (41), and suppresses cytotoxic immune activity, contributing to a fibrotic and immunosuppressive microenvironment (42). Dual blockade of IL-6 and PD-L1 has been shown to enhance IFN-γ^+^ Th1 polarization and NK cell-mediated antitumor immunity, improving survival in murine PDAC models (43). Three IL-6 antagonists, siltuximab, tocilizumab, and sarilumab, are currently approved for clinical use. Preclinical studies support the synergistic potential of combining IL-6 blockade with ICIs (37), providing a strong rationale for clinical translation in PDAC. Although IL-6 inhibition alone has shown limited success in pancreatic cancer, likely due to compensatory pathways, combination strategies with PD-1/PD-L1 inhibitors may yield synergistic benefits. Our data, suggesting a potential role for IL-6 signaling in modulating the response to chemo-immunotherapy in PDAC, are further contextualized by several ongoing clinical trials actively exploring this combination strategy (NCT05704634, NCT04191421, NCT06470971, NCT04729959, NCT05428007, NCT03999749, NCT04940299). These ongoing efforts underscore the significant clinical interest in mitigating cytokine-mediated resistance to ICIs and support the biological rationale of our findings. The outcomes of these trials will be pivotal in determining the future clinical applicability of combining IL-6 pathway inhibition with immunotherapy regimens in PDAC and other cancers.

Pivotal trials like NAPOLI-3 and PRINCE are essential for establishing the clinical efficacy of novel regimens against standard chemotherapy, their primary focus is on comparing OS and progression-free survival PFS. Our integrated multi-omics approach—correlating clinical outcomes with flow cytometry, multiplex immunofluorescence, and whole transcriptome sequencing—provides a unique layer of insight that large-scale trials typically do not. Where these large studies often rely on single biomarkers (e.g., dMMR/MSI-H status), and limited analysis of the immune landscape. We conducted an unbiased discovery of novel cellular, spatial, and genomic correlates. For instance, we identified that a suppressive systemic milieu (high CD45^-^ CD64^+^ cells) and an activated tumor microenvironment (CD137^+^ CD8^+^ T cells) are key determinants of outcome, and we nominated novel candidate biomarkers like HPX and RFXAP that are associated with positive prognosis. While previous large-scale trials have primarily focused on establishing clinical efficacy, our study is uniquely positioned to provide a deep mechanistic understanding of the tumor immune microenvironment (TIME) and peripheral immune response in patients receiving chemo-immunotherapy combinations. Our integrated use of flow cytometry for real-time peripheral immune monitoring, mIF for spatial context within the tumor, and WTSS for unbiased genomic discovery on patient-derived samples is, to our knowledge, one of the most comprehensive multi-omics assessments conducted alongside a clinical study in advanced PDAC. This approach allowed us to move beyond reporting clinical endpoints and to identify correlative biomarkers, generate actionable hypotheses, and decipher mechanism. The WTSS data provides a rich resource for understanding the transcriptional programs associated with response and resistance, offering clues for overcoming immunotherapy resistance in PDAC.

Thus, the fundamental added value of our findings lies in translating clinical outcomes into biological insights. We move beyond reporting *what* the outcome was to begin explaining *why* it occurred. While confirmatory Phase III trials are essential, our study serves a different but critical purpose: it functions as a hypothesis-generating translational science component. We provide the biological rationale and candidate biomarkers that could inform the design of future trials, such as stratifying patients based on HPX expression or combining immunotherapy with agents that target the IL-6 pathway.

## Limitations

This study has several limitations that must be considered. 1. The primary survival analysis was based on PFS, and mature OS data were not available at the time of this analysis, the definitive assessment of the survival benefit of this combination regimen awaits the final analysis of OS data from larger, randomized controlled trials; 2. Different PD-1/PD-L1 inhibitors were pooled for analysis due to sample size constraints, and the potential for subtle differences in efficacy between specific agents cannot be ruled out; 3. The most significant is the relatively small overall sample size (n=52), and particularly the very small sub-cohort (n=6) available for WTSS analysis. While our cohort was carefully selected, the limited sample size reduces the statistical power to detect small to moderate effect sizes. This increases the probability of Type II errors, meaning that some true associations or differences might have been overlooked. Furthermore, the generalizability of our findings, especially those from the WTSS subgroup, may be constrained. These findings should therefore be interpreted as preliminary and hypothesis-generating, requiring validation in larger, independent cohorts; 4. We acknowledge the potential for batch effects in the generation of our omics data, particularly the WTSS. To mitigate this, RNA extraction, library preparation, and sequencing were performed using standardized, automated protocols to minimize technical variability. During bioinformatic analysis, we employed rigorous quality control metrics and utilized statistical methods to visually inspect for any batch-related clustering. No significant batch effects were detected that correlated with the experimental groups. Nevertheless, we cannot entirely rule out subtle undetected technical variation, and this is a common limitation of all omics studies; 5. The multi-omics and immune profiling data in this study are derived from a single time point prior to treatment initiation. This cross-sectional design, while providing a comprehensive baseline profile, inherently limits our ability to track the evolution of the immune landscape under therapeutic pressure, and identify on-treatment pharmacodynamic biomarkers of response. The lack of longitudinal immune monitoring is a key limitation. Future studies incorporating serial liquid biopsies and on-treatment tumor biopsies would be invaluable to understand the dynamic mechanisms of efficacy and resistance, and to differentiate between predictive and prognostic biomarkers.

## Conclusion

In conclusion, our findings suggest that immunotherapy combined with chemotherapy, particularly gemcitabine-based first-line treatment, may improve outcomes in PDAC patients. Multi-omics analyses have delineated distinct immune and molecular features associated with treatment response, providing novel insights for developing combination strategies and identifying predictive biomarkers. Further research is needed to fully elucidate the immune landscape, tumor microenvironment heterogeneity, and molecular mechanisms underlying treatment response, which may ultimately improve outcomes for patients with this challenging disease.

## Data Availability

The data presented in the study are deposited in the SRA repository, accession number PRJNA1292091.
