# Supplementary material for: Multi-omics analyses inform mechanisms of immunotherapy response in pancreatic cancer
Source: Front Immunol. 2025 Oct 2;16:1673098. doi: 10.3389/fimmu.2025.1673098 (PMC12528169; doi:10.3389/fimmu.2025.1673098)
Supplement: Supplementary file 1 [file DataSheet1.docx]

Supplementary Materials for

Multi-omics analyses inform mechanisms of immunotherapy response in pancreatic cancer

Mengyao Wu, Ge Li, Yecheng Li, Kai Chen, Mengdan Xu, Dapeng Li, Caihua Xu, Meng Shen^§^, Wei Li^§^, Jinming Cao^§^

Correspondence to: Jinming Cao (caojinming@suda.edu.cn), Wei Li ([liwei10@suda.edu.cn](mailto:liwei10@suda.edu.cn)) or Meng Shen (doctor_shenm@163.com)

**This PDF file includes:**

Supplementary Fig 1 to 2

Supplementary Table 1 to 2

**
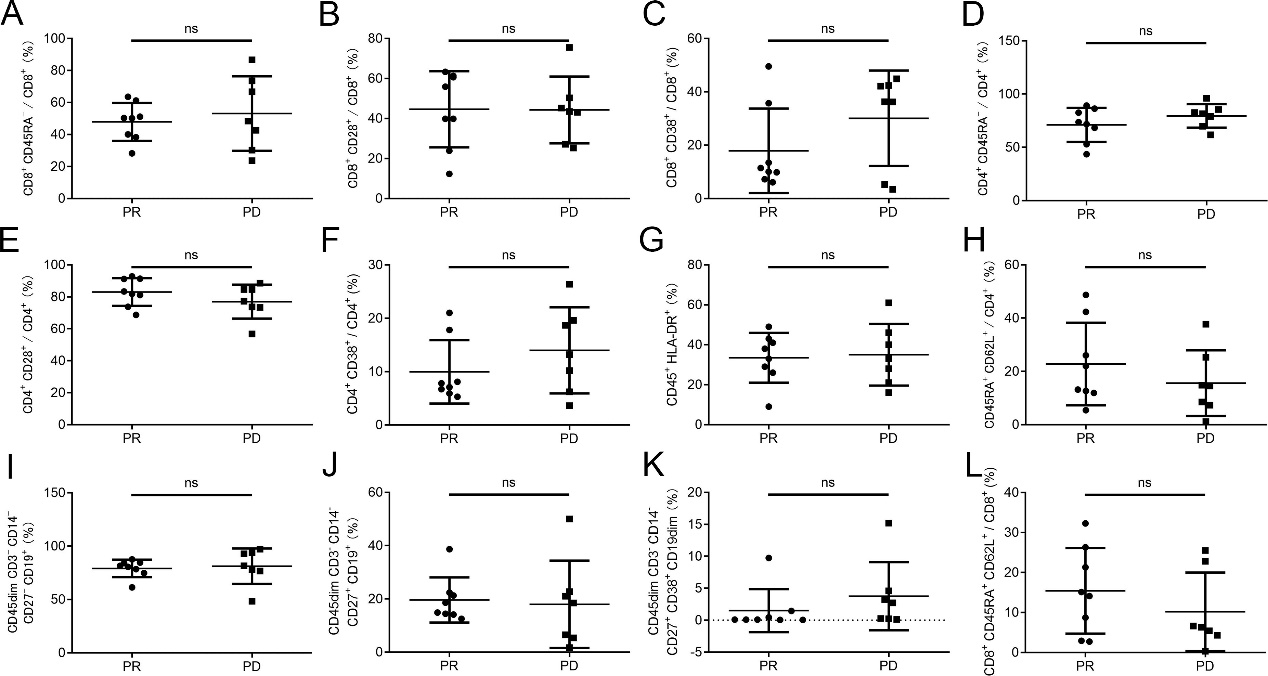
**

**Supplementary Figure 1.** The immune phenotype of peripheral blood in PR and PD patients at baseline. A. Rate of CD8+ CD45RA- / CD8+ leukocytes in PR and PD patients. B. Rate of CD8+ CD28+ / CD8+ leukocytes in PR and PD patients. C. Rate of CD8+ CD38+ /CD8+ leukocytes in PR and PD patients. D. Rate of CD4+ CD45RA- / CD4+ leukocytes in PR and PD patients. E. Rate of CD4+ CD28+ / CD4+ in PR and PD patients. F. Rate of CD4+ CD38+ / CD4+ leukocytes in PR and PD patients. G. Percentage of CD45+ HLA-DR+ leukocytes in PR and PD patients. H. Rate of CD45RA+ CD62L+ / CD4+ leukocytes in PR and PD patients. I. Percentage of CD45dim CD3- CD14- CD27- CD19+ leukocytes in PR and PD patients. J. Percentage of CD45dim CD3- CD14- CD27+ CD19+ leukocytes in PR and PD patients. K. Percentage of CD45dim CD3- CD14- CD27+ CD38+ CD19dim leukocytes in PR and PD patients. L. Rate of CD8+ CD45RA+ CD62L+ / CD8+ leukocytes in PR and PD patients. All data shown as the mean ± SD; ns, not significant.

**
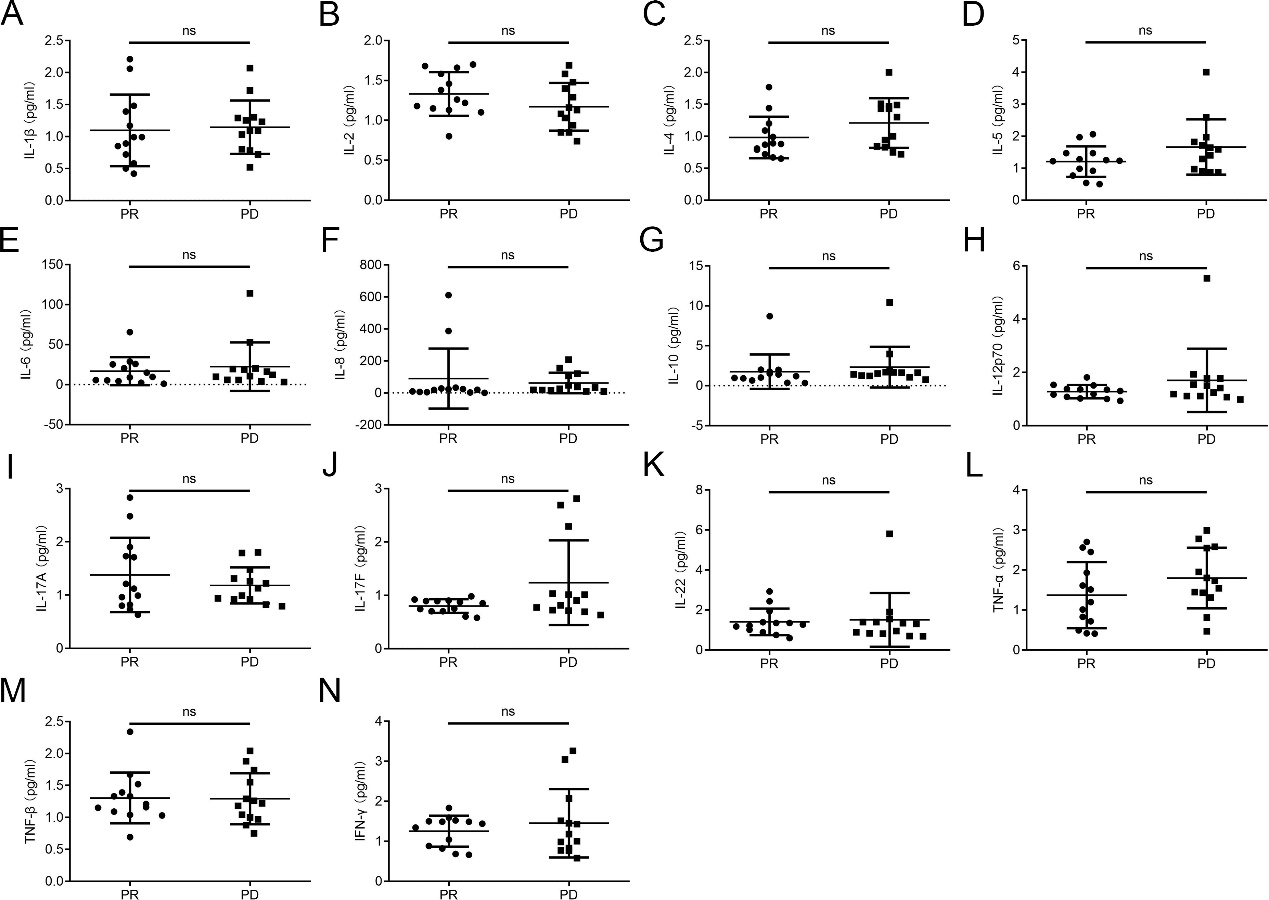
Supplementary Figure 2.** Cytokine levels of peripheral blood in PR and PD patients at baseline. A. IL-1β level of plasma in PR and PD patients. B. IL-2 level of plasma in PR and PD patients. C. IL-4 level of plasma in PR and PD patients. D. IL-5 level of plasma in PR and PD patients. E. IL-6 level of plasma in PR and PD patients. F. IL-8 level of plasma in PR and PD patients. G. IL-10 level of plasma in PR and PD patients. H. IL-12p70 level of plasma in PR and PD patients. I. IL-17A level of plasma in PR and PD patients. J. IL-17F level of plasma in PR and PD patients. K. IL-22 level of plasma in PR and PD patients. L. TNF-α level of plasma in PR and PD patients. M. TNF-β level of plasma in PR and PD patients. N. IFN-γ level of plasma in PR and PD patients. All data shown as the mean ± SD; ns, not significant.

**Supplementary Table 1.** Baseline characteristics across gemcitabine-, fluorouracil-, and other-based regimens

| Characteristic | Gemcitabine, *n* | Fluorouracil, *n* | Others, *n* | *P* |
| --- | --- | --- | --- | --- |
| Age |  |  |  | 0.277 |
| < 66 | 14 | 10 | 1 |  |
| ≥ 66 | 14 | 8 | 5 |  |
| Sex |  |  |  | 0.122 |
| Male | 21 | 8 | 4 |  |
| Female | 7 | 10 | 2 |  |
| Treatment lines |  |  |  | 0.008^**^ |
| 1 | 21 | 6 | 2 |  |
| ≥2 | 7 | 12 | 4 |  |
| Immunotherapy received |  |  |  |  |
| PD-1 | 22 | 13 | 6 | 0.495 |
| PD-L1 | 6 | 5 | 0 |  |

**Supplementary Table 2.** Multivariable Cox regression of baseline characteristics

| Variable | Adjusted Hazard Ratio  (95% CI) | *P* |
| --- | --- | --- |
| Age (< 66 vs. ≥ 66) | 1.056 (0.546-2.041) | 0.872 |
| Sex (Male vs. Female) | 0.944 (0.440-2.024) | 0.882 |
| Previous treatment regimens (1 vs. ≥ 2) | 1.638 (0.813-3.302) | 0.167 |
| Immunotherapy received (PD-1 vs. PD-L1) | 1.137 (0.516-2.509) | 0.750 |
| Chemotherapy regimens |  |  |
| Fluorouracil vs. Gemcitabine | 2.163 (0.687-6.813) | 0.187 |
| Others vs. Gemcitabine | 3.635 (0.968-13.656) | 0.056 |
